# Clinical validity and diagnostic accuracy of the Maslach Burnout Inventory-Student Survey in Sri Lanka

**DOI:** 10.1186/s12955-018-1048-y

**Published:** 2018-11-20

**Authors:** Nuwan Darshana Wickramasinghe, Devani Sakunthala Dissanayake, Gihan Sajiwa Abeywardena

**Affiliations:** 1 Department of Community Medicine, Faculty of Medicine and Allied Sciences, Rajarata University of Sri Lanka, Saliyapura, 50008 Sri Lanka; 2 Department of Community Medicine, Faculty of Medicine, University of Peradeniya, Peradeniya, 20400 Sri Lanka; 3 Teaching Hospital-Kandy, Kandy, 20000 Sri Lanka

**Keywords:** Student burnout, MBI-SS, Clinical validity, Diagnostic accuracy, Collegiate cycle, Sri Lanka

## Abstract

**Background:**

Absence of context-specific clinically validated cut-off values for assessing burnout as a dichotomous phenomenon has hindered the progress of student burnout research with regard to quantifying the magnitude of the problem. Hence, the present study was aimed at developing clinically validated cut-off values and evaluating the diagnostic accuracy of the Sinhala translation of the 15-item Maslach Burnout Inventory-Student Survey (MBI-SS) in assessing burnout among collegiate cycle students in Sri Lanka.

**Methods:**

This prospective validation study was conducted among 194 grade thirteen students in the Kurunegala district, Sri Lanka. Clinically validated cut-off values for the subscale scores of the MBI-SS test was developed by computing ROC curves, using the clinical diagnosis made by the Consultant Psychiatrist as the reference standard. Diagnostic accuracy of the MBI-SS test results based on “exhaustion+ 1” criterion was assessed comparing with the results of the clinical diagnosis.

**Results:**

The clinically validated cut-off values for the exhaustion, cynicism and reduced professional efficacy subscale scores were 12.5, 7.5 and 10.5 for the respectively. The sensitivity, specificity, positive and negative predictive values of the Sinhala translation of the 15-item MBI-SS were 91.9% (95% CI = 82.5–96.5%), 93.2% (95% CI = 87.5–96.4%), 86.4% (95% CI = 76.1–92.7%) and 96.1% (95% CI = 91.2–98.3%) respectively. The positive and negative likelihood ratios were 13.48 (95% CI = 7.15–25.44) and 0.09 (95% CI = 0.04–0.20) respectively.

**Conclusions:**

By using the clinically validated cut-off values for the subscale scores and based on the “exhaustion + 1” criterion, the Sinhala translation of the 15-item MBI-SS could be effectively used as a screening tool to assess burnout among collegiate cycle students. The study findings broaden the global evidence base pertaining to validated cut-off values of the MBI-SS.

## Background

Against the backdrop of ever-increasing socio-economic, political and cultural complexities intermingled with educational environments having higher expectations and demands, burnout has emerged as a major problem negatively affecting the well-being of the student populations [1]. The most widely accepted definition of student burnout describes student burnout as, “a three-dimensional syndrome that is characterised by feelings of exhaustion due to the demands of studying, a cynical attitude of withdrawal and detachment, and reduced professional efficacy regarding academic requirements” [2].

Though various study instruments have been used to assess burnout among student populations [3–5], the first reported literature pertaining to the invention of a specific measure to assess student burnout is the invention of Maslach Burnout Inventory-Student Survey (MBI-SS) by Schaufeli et al. [2], which is a self-report measure modified from the MBI-General Survey and targeted at identifying the three subscales of burnout, viz., exhaustion (EX), cynicism (CY) and professional efficacy (PE). Since then, MBI-SS has been cited as the most widely used research instrument to assess burnout in different student populations across the globe [6–8].

The validity and the reliability of different translated versions of the MBI-SS have been established in numerous settings [2, 8–13]. MBI-SS, due to its brevity, ease of administration and sound psychometric properties, could be used as an effective screening tool for the assessment of burnout among different student populations.

Even though the multidimensionality of burnout is widely acknowledged [14–16], there are theoretical and practical reasons to consider burnout as a single construct such as estimating the prevalence of burnout in a sample [15] and diagnostic purposes in medical practice [17]. However, the unidimensional approach is criticized due to a number of reasons including the considerable loss of information by combining the dimensions, which have complex associations between themselves and with other variables [15, 18], and the possible difference in the role of the dimensions in different phases in the process of burnout. [15, 19].

The main obstacle in defining burnout as a dichotomous phenomenon in epidemiological research is the absence of ubiquitous clinically validated cut-off values to be used for the psychometric assessment tools including MBI-SS. The fact that MBI produces continuous scores on multiple dimensions of burnout, poses the challenge of translating the continuous scores of the research measure into a dichotomous burnout classification [14]. In order to translate the continuous scores of the research measure into a dichotomous classification by establishing cut-off points, an external criterion such as independent burnout diagnosis is often used. In relation to that, clinical diagnosis based on the 10th revision of the International Statistical Classification of Diseases and Related Health Problems (ICD-10) “work-related neurasthenia” was cited as the gold standard of diagnosis of burnout [14, 16, 17, 20–22]. Based on the clinical diagnostic criteria, clinically validated cut-off scores for each of the three MBI-SS subscales can be established.

With a view of reflecting the multidimensional nature of burnout without formulating a composite score, the “exhaustion+ 1” criterion is recommended for research [14–16, 22, 23]. According to this criterion, an individual is considered to be having burnout when he or she has a “high” score on EX in combination with a “high” score on either of the CY or rPE subscales of MBI. This “high” score can be determined by the clinically validated cut-off values.

In comparison to the wealth of research conducted among different student populations pertaining to burnout across the globe, the published literature on the topic in the South Asian context is scanty with no published literature on burnout among Sri Lankan school students. In Sri Lanka, the collegiate cycle in the education system (consists of grade twelve and grade thirteen) leads to the General Certificate of Examination Advanced Level, which is the national level selection examination for state university admissions and evidence suggests that there is a high prevalence of mental health problems among collegiate cycle students [24–26]. In the light of this evidence, it is of utmost importance to explore the concept of student burnout; yet, the main hindering factor for this research vacuum is the absence of validated instruments to assess burnout. Furthermore, for the purpose of assessing the prevalence of burnout in target populations context-specific cut-off values need to be developed to translate the continuous scores of the research instrument into a dichotomous burnout classification.

Against this background, the present study was designed with the objectives of developing clinically validated cut-off values for the Sinhala version of the 15-item MBI-SS subscale scores and evaluating the diagnostic accuracy of the Sinhala version of the 15-item MBI-SS in assessing burnout among collegiate cycle students in Sri Lanka.

## Methods

The findings of the present study are reported following the Standards for Reporting of Diagnostic Accuracy Studies (STARDS 2015) guidelines [27].

### Study design and setting

This prospective validation study was conducted in a selected educational division in the Kurunegala district, North Western province, Sri Lanka from January 2015 to March 2015. In the selected educational division, three schools were selected based on the logistic feasibility to conduct clinical interviews by the Consultant Psychiatrist, ease of accruing a relatively large number of students on a given date and having satisfactory infrastructure facilities in the schools to arrange suitable places for data collection and conducting clinical interviews. Furthermore, particular emphasis was paid in selecting both male and female students studying in all four subject streams in the collegiate cycle, viz., Science, Arts, Commerce and Technology.

Ethical clearance to conduct the study was obtained from the Ethics Review Committee of the Faculty of Medicine and Allied Sciences, Rajarata University of Sri Lanka. Prior to data collection, administrative clearance was obtained from the Provincial Director of Education, North Western province and the principals of the selected three schools. The dates for data collection in different schools were selected according to the logistic convenience of the schools in order to minimise the disturbance to the routine academic and other endeavors.

### Participants

In this single-gate study, all grade thirteen students studying in the selected study setting at the time of the study were considered as eligible participants. The sample size required to conduct the validation study was calculated by using the standard sample size calculation formula [28] for an anticipated sensitivity/specificity of 90%, a confidence level of 95% and the level of precision of 15% and the resultant number of students with burnout to be enrolled in the study was 62. All eligible participants were identified based on the data available in the classroom registers and were recruited for the study consecutively upon their informed written consent and the Consultant Psychiatrist interviewed a total of 194 grade thirteen students.

### Measures

#### Sinhala version of the 15-item MBI-SS

In this validation study, the Sinhala version of the 15-item MBI-SS was used as the index test. MBI-SS has been cited as the most widely used research instrument to assess burnout in different student populations across the globe [6–8]. Moreover, the Sinhala version of the 15-item MBI-SS was found to be a valid and a reliable instrument to assess the burnout status among collegiate cycle students in Sri Lanka [29]. The construct validity of the MBI-SS was assessed using multi-trait scaling analysis and confirmatory factor analysis, while reliability was assessed using internal consistency and test-retest reliability. Multi-trait scaling analysis has revealed that item 13 has poor psychometric properties. In the confirmatory factor analysis, a three-factor model of the Sinhala version of the 15-item MBI-SS (with deleted item 13) has emerged as an acceptable fitting model with a combination of absolute, relative and parsimony fit indices reaching desired threshold values. Internal consistency assessment has shown that all three subscales having high Cronbach’s α coefficient values of 0.84, 0.87 and 0.88 for EX, CY and rPE subscales respectively. The test-retest reliability correlation coefficients were 0.86, 0.91 and 0.89 for the EX, CY and rPE subscales respectively (*p* < 0.001) [29].

The Sinhala version of the MBI-SS, which is a self-administered questionnaire, consists of 15 items representing the three dimensions of student burnout. Out of the total 15 items, five items are targeted at identifying EX, four items are targeted at identifying CY and six items are targeted at identifying PE. The frequency in which the respondents experience feelings related to each dimension was assessed using a seven-point, fully anchored response format. The rating scale ranges from 0 (never) to 6 (every day). High scores on EX and CY while low scores on PE are indicative of burnout. The minimum attainable scores for all the three subscales are 0, where as the maximum attainable scores for EX, CY and PE subscales are 30, 24 and 36 respectively.

#### Clinical interview by a consultant psychiatrist

The clinical criteria for the diagnosis of work related neurasthenia according to the ICD 10 classification has been used in the clinical diagnosis of burnout as well as in research as the “gold standard” for detecting burnout [14, 15, 21, 22]. Thus, the clinical assessment of burnout by a Consultant Psychiatrist was conducted as the reference standard in this validation study. The clinical assessment was based on the ICD classification and according to the ICD 10 classification, the condition is characterised by increased fatigue after minor mental effort often associated with some decrease in performance or coping efficiency in daily tasks. The mental fatiguability is described as an unpleasant intrusion of distracting associations or recollections, difficulty in concentrating, and generally inefficient thinking. Another common presentation includes feelings of bodily or physical weakness and exhaustion after only minimal effort, accompanied by a feeling of muscular aches and pains and inability to relax. Furthermore, unpleasant physical feelings such as dizziness, tension headaches, sleeping disturbances and feelings of general instability are given as common associations [30].

### Procedure

Upon the informed written consent, each participant was given a unique serial number in order to maintain anonymity and for the future identification in the study. All participants were given the Sinhala version of the 15-item MBI-SS. Confidentiality of data collected was adhered to strictly and the anonymity of the participants was maintained.

The clinical interview was conducted in a selected classroom ensuring privacy and confidentiality. An experienced Consultant Psychiatrist, who was blinded to the scores of the MBI-SS, interviewed each participant individually and the clinical assessment was made in relation to the burnout status.

The scores of the three subscales of the MBI-SS for each participant were computed according to the instructions provided in the MBI manual [31] and the investigator was blinded to the results of the clinical assessment of the participants. As high scores on EX and CY subscales are indicative of burnout, whereas low scores on PE subscale is indicative of burnout, reverse scores of PE (rPE) were calculated for the analysis.

### Data analysis

Clinical validity of the MBI-SS was assessed by comparing the three subscale scores of the MBI-SS with the clinical diagnosis made by the Consultant Psychiatrist as the gold standard. Receiver operating characteristic (ROC) curves were computed for different cut-off values of the MBI-SS subscales, by using SPSS version 17.0.

In determining optimal cut-off values, two methods were used. The square of the distance (d^2^) between the point on the upper left corner of ROC space and any point on ROC curve was calculated using a standard formula [32]. The lowest distance from the curve was regarded as the optimal cut-off value. In addition, the Youden index which reflects the vertical distance of ROC curve from the point on diagonal line was calculated for different cut-off values using a standard formula [32]. The corresponding value, which maximises the Youden index, was considered as the optimal cut-off value. These optimal cut-off values were regarded as the clinically validated cut-off values for each of the subscale of the MBI-SS.

Since “exhaustion+ 1” criterion is the most acceptable method enabling the multidimensional continuous measure of MBI to be translated into a dichotomy in order to diagnose burnout [14, 15, 21, 22], based on the clinically validated cut-off values for the three subscales, a student who is having a high score on EX in combination with a high score on either of the CY or rPE subscale was categorised as having burnout. A high score in each subscale was considered as any score equal or more than the developed cut-off value for the respective subscale.

Based on the above categorisation, the sensitivity, specificity, Positive Predictive Value (PPV), Negative Predictive Value (NPV), Positive Likelihood Ratio (LR+) and Negative Likelihood Ratio (LR-) of MBI-SS in assessing burnout status were calculated [33].

## Results

### Descriptive statistics

The study sample consisted of 194 grade thirteen students and all participants completed the Sinhala version of the MBI-SS and underwent the clinical assessment by the Consultant Psychiatrist. The response rate was 100%.

The proportion of female students in the sample was 55.2% (*n* = 107). The majority (40.2%) of students were studying in Science stream (*n* = 78). The mean age of the sample was 18.3 years (SD = 0.43 years). Distribution of the sample by sex and subject stream is given in Table 1.

**Table 1:** Distribution of the sample according to sex and subject stream (*n* = 194)

Characteristic	Number	Percentage (%)	Cumulative Percentage (%)
Sex			
Female	107	55.2	55.2
Male	87	44.8	100.0
Subject stream			
Science	78	40.2	40.2
Arts	60	30.9	71.1
Commerce	41	21.2	92.3
Technology	15	7.7	100.0
Total	194	100.0	

The observed values of the EX subscale ranged from 0 to 26, 0 to 22 of the CY subscale and 0 to 30 of the rPE subscale. The mean total scores of the three subscales were 11.66 (SD = 6.29), 6.89 (SD = 5.92) and 9.93 (SD = 7.61) for the EX, CY and rPE subscales respectively.

Independent sample t-test was conducted to compare the mean total scores of MBI-SS subscales between female and male students. The difference in mean total scores of all three MBI-SS subscales were not statistically significant between the two groups (t (192) = − 0.223, *p* = 0.824; t (192) = − 0.497, *p* = 0.620 and t (192) = − 1.270, *p* = 0.206 for EX, CY and rPE subscales respectively).

The final sample consisted of 62 (32.0%) students who were categorised as having burnout and 132 (68.0%) students without burnout according to the clinical assessment conducted by the Consultant Psychiatrist.

Developing the clinically validated cut-off values for MBI-SS subscale scores.

**Fig. 1:**
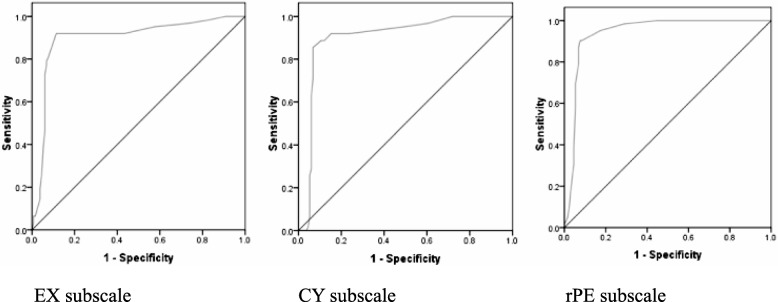
Receiver operating characteristic curves for three subscale scores against clinical diagnosis. (EX: Exhaustion; CY: Cynicism, rPE: reversed Professional Efficacy)

ROC curves were computed using the total scores of MBI-SS subscales as the test variable and the clinical diagnosis of burnout as the criterion variable. Figure 1 shows the ROC curves plotted for different cut-off values of the three subscale scores, giving the sensitivity against different values for (1-specificity).

According to the statistics of the area under the curve, the overall ability of the test to discriminate between those with burnout and those without burnout by EX subscale is 89.7% (95% CI = 84.3–95.1%). The corresponding values for the subscales of CY and rPE are 93.9% (95% CI = 90.4–97.4%) and 90.0% (95% CI = 85.0–95.1%) respectively.

Table 2 shows the sensitivity, specificity, distance from the curve and the calculated Youden index for different cut-off values of the three subscale scores.

**Table 2:** Sensitivity, specificity, distance from curve and Youden index for different cut-off values of the EX, CY and rPE subscales

Subscale	Cut-off Value ^*^	Sensitivity	Specificity	(1 – Specificity)	Distance from Curve	Youden Index
EX	8.50	0.919	0.568	0.432	0.439	0.487
	9.50	0.919	0.712	0.288	0.299	0.631
	10.50	0.919	0.795	0.205	0.220	0.714
	11.50	0.919	0.841	0.159	0.178	0.760
	12.50	0.919	0.886	0.114	0.139	0.805
	13.50	0.806	0.924	0.076	0.208	0.730
	14.50	0.790	0.932	0.068	0.221	0.722
	15.50	0.774	0.932	0.068	0.236	0.706
	16.50	0.726	0.939	0.061	0.281	0.665
CY	3.50	1.000	0.553	0.447	1.000	0.553
	4.50	0.984	0.712	0.288	1.000	0.696
	5.50	0.952	0.826	0.174	1.000	0.778
	6.50	0.903	0.917	0.083	0.128	0.820
	7.50	0.903	0.924	0.076	0.123	0.827
	8.50	0.871	0.932	0.068	0.146	0.803
	9.50	0.855	0.932	0.068	0.160	0.787
	10.50	0.839	0.932	0.068	0.175	0.771
	11.50	0.790	0.932	0.068	0.221	0.722
rPE	6.50	0.935	0.636	0.364	0.369	0.571
	7.50	0.919	0.765	0.235	0.248	0.684
	8.50	0.919	0.848	0.152	0.172	0.767
	9.50	0.887	0.879	0.121	0.166	0.766
	10.50	0.887	0.894	0.106	0.155	0.781
	11.50	0.855	0.932	0.068	0.160	0.779
	12.50	0.839	0.932	0.068	0.175	0.771
	13.50	0.790	0.932	0.068	0.221	0.722
	14.50	0.710	0.932	0.068	0.298	0.642

*The cut-off values for EX range from 0 to 26, CY range from 0 to 22 and rPE range from 0 to 30 and only a selection of scores are reported without the extreme values

According to Table 2, the cut-off value was taken as 12.50 or above for the EX subscale score. At this cut-off value, the EX subscale score had a sensitivity of 91.9% (95% CI = 82.2–97.3%) and a specificity of 88.6% (95% CI = 82.0–93.5%). The optimum cut-off value was taken as 7.50 for the CY subscale score of the MBI-SS. At this cut-off value, the CY subscale score had a sensitivity of 90.3% (95% CI = 80.1–96.4%) and the specificity of 92.4% (95% CI = 86.5–96.3%). The rPE subscale score was found to have a sensitivity of 88.7% (95% CI = 78.1–95.3%) and a specificity of 89.4% (95% CI = 82.8–94.1%) at an optimal cut off value of 10.50.

In addition to the above cut-off values, optimum cut-off values were computed for female (*n* = 107) and male (*n* = 87) students separately. For female students, the optimum cut-off values for the EX, CY and rPE subscales were 12.5 (corresponding sensitivity 85.7% and specificity 88.9%), 7.0 (corresponding sensitivity 82.9% and specificity 93.1%) and 10.5 (corresponding sensitivity 80.0% and specificity 91.7%) respectively. For male students, the optimum cut-off values for the EX, CY and rPE subscales were 12.5 (corresponding sensitivity 100.0% and specificity 88.3%), 8.5 (corresponding sensitivity 100.0% and specificity 91.7%) and 12.5 (corresponding sensitivity 100.0% and specificity 91.7%) respectively.

#### Diagnostic accuracy of the Sinhala version of 15-item MBI-SS

**Fig. 2:**
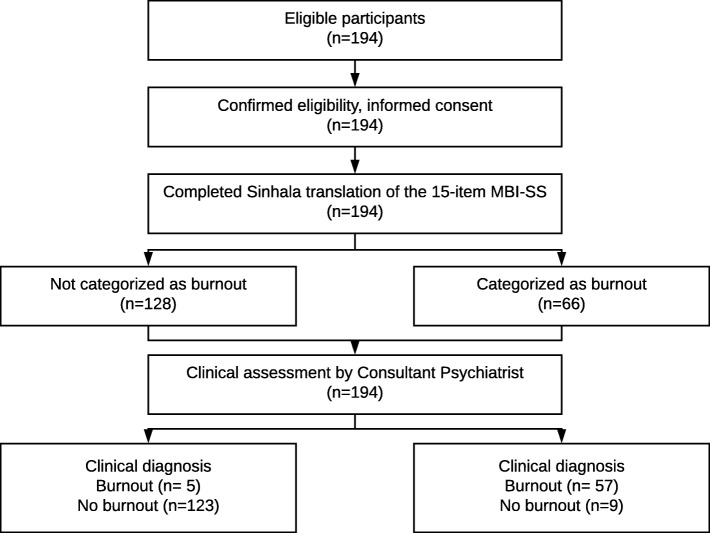
Flow diagram of participants through the study (The Consultant Psychiatrist was unaware of the results of the MBI-SS scores.)

Figure 2 illustrates the flow of participants through the study (developed retrospectively).

Table 3 summarises the categorisation of the participants of the validation study according to clinical diagnosis against the diagnosis made by the clinically validated cut-off values for MBI-SS subscales.

**Table 3:** Comparison of clinical diagnosis against the diagnosis made by the clinically validated cut-off values for the MBI-SS subscales for 194 grade thirteen students

		MBI-SS Diagnosis				Total	
		Burnout		Not burnout			
		n	%	n	%	n	%
Clinical Diagnosis	Burnout	57	91.9	5	8.1	62	100.0
	Not burnout	9	6.8	123	93.2	132	100.0
	Total	66	34.0	128	66.0	194	100.0

Indicators of diagnostic accuracy of the MBI-SS in assessing burnout status were calculated and the results are summarised in Table 4. All calculated indicators, except PPV, were higher than 90%. Based on the sensitivity and specificity values, LR+ and LR- for the test were computed as 13.48 (95% CI = 7.15–25.44) and 0.09 (95% CI = 0.04–0.20).

**Table 4:** Summary of indicators of diagnostic accuracy of the MBI-SS assessment of burnout status in 194 grade thirteen students

Indicator	Formula	Value	95% CI
Sensitivity	TP / (TP + FN)	91.9%	82.5–96.5%
Specificity	TN / (TN + FP)	93.2%	87.5–96.4%
PPV	TP / (TP + FP)	86.4%	76.1–92.7%
NPV	TN / (TN + FN)	96.1%	91.2 - 98.3%
LR+	Sensitivity/(1-Specificity)	13.48	7.15–25.44
LR-	(1-Sensitivity)/Specificity	0.09	0.04–0.20

95% *CI*: 95% Confidence Interval; *FN*: False Negatives; *FP*: False Positives; *LR+*: Positive Likelihood Ratio; *LR-*: Negative Likelihood Ratio; *PPV*: Positive Predictive Value; *NPV*: Negative Predictive Value; *TN*: True Negatives; *TP*: True Positives.

## Discussion

The concept of student burnout has been explored across different student populations representing varying educational contexts. However, the novelty of the concept and the absence of a proper assessment tool have hindered the exploration of the concept in many of the South Asian countries including Sri Lanka. Furthermore, the absence of ubiquitous clinically validated cut-off values for the assessment of burnout as a dichotomous phenomenon has hindered the progress of burnout research assessing the magnitude of the problem.

In the present study, MBI-SS was selected as the index test, which has been widely cited as the most commonly used burnout measure across the globe [6–8]. Moreover, the validity and the reliability of the Sinhala version of the 15-item MBI-SS have been established in the Sri Lankan context [29]. The Sinhala version of the 15-item MBI-SS has omitted item 13 from the original 16-item MBI-SS due to its poor psychometric properties [29] and similarly, this item was identified as having poor psychometric properties by some other researchers largely owing to its ambivalent nature [2, 8–10, 34, 35]. Thus, the usage of the 15-item MBI-SS as the index test is justifiable.

In keeping with the widely accepted method employed by many researchers [14, 20, 21], psychiatric clinical diagnosis based on ICD-10 work-related neurasthenia was used as the gold standard diagnosis of burnout in appraising the criterion validity in the present study. The Consultant Psychiatrist was blinded to the responses of the MBI-SS by each participant at the time of clinical interview. Hence, there was no bias in the clinical assessment of burnout based on the MBI-SS scores. Furthermore, the clinical assessment in the present study is free of inter-observer variation, given that a single experienced Consultant Psychiatrist has conducted all clinical interviews.

The “exhaustion+ 1” criterion, which was recommended by many researchers [14, 15, 21, 22], was used as the method of categorising the participants as having burnout by using the MBI-SS scores. Hence, the most appropriate method of dichotomous categorisation of burnout by using MBI was employed in the present study.

In the present study, all participants have undergone both index test and reference standard and there was no time interval between index test and reference standard, allowing the target condition to change.

Previous studies using a 15-item MBI-SS to assess burnout among medical students have used either the 66th percentile [35] or the 75th percentile [34] values of the subscale score distribution as the cut-off values to classify burnout status. In both of the studies, the reported cut-off value of the EX subscale was equivalent to a score greater than 14 [34, 35]. The cut-off value of the CY subscale was equivalent to a score greater than 6 [35] and a score greater than 9 [34]. Even though the cut-off values could not be directly compared across the studies, mainly due to the differences in the study methodology, the reported cut-off values are not drastically different from the values of the present study.

Furthermore, it is important to note that there are no statistically significant differences in the mean total scores of MBI-SS subscales between female and male students and the computed cut-off values for female and male students are similar to those of the total study sample.

In the present study, the “exhaustion+ 1” criterion used for the MBI-SS, had revealed a sensitivity of 91.9% and a specificity of 93.2%. Both these indicators were highly satisfactory. Thus, the ability of the test to correctly identify students with clinical burnout as well as the ability to establish the absence of clinical burnout in students who are indeed not having clinical burnout is high. Furthermore, the predictive values for MBI-SS were also highly satisfactory with a PPV of 86.4% and NPV of 96.1%. In addition, the interval estimates of these indicators of diagnostic accuracy were fairly narrow indicating the sample adequacy.

With regard to assessing the diagnostic accuracy, likelihood ratios are considered as the best indicators, since both specificity and sensitivity values are used to calculate the likelihood ratios [36–38]. The LR+ is considered as the best indicator for ruling-in diagnosis of interest and the LR- is considered as the best indicator for ruling-out the diagnosis of interest. In the present study, the LR+ is high indicating that the likelihood of a student having burnout has increased by approximately thirteen-fold given the positive test result (13.48, 95% CI = 7.15–25.44). Similarly, the LR- of the present study indicates how much less likely the negative test result is to occur in a student with burnout than in a student without burnout and the ratio is as low as 0.09 (95% CI = 0.04–0.20).

### Limitations

Despite possible measures taken to overcome the foreseeable limitations during the planning and implementation stages of the present study, the study is with some limitations inherent to the methodology and limitations pertaining to the interpretation of study findings. The study sample primarily consisted of grade thirteen students from a selected educational division and the sample was recruited based on reasons related to logistic feasibility. Thus, the generalisability of the validated cut-off values to other student populations should be done with caution, considering the variations in educational and cultural contexts.

Since a single Consultant Psychiatrist has conducted all clinical interviews, the inter-observer reliability could not be computed. As the clinically validated cut-off values for index test were developed based on the comparison with the clinical reference standard, there is a possibility of overestimation of the diagnostic accuracy of the MBI-SS.

In assessing the construct validity of the MBI-SS, convergent or discriminant validity with the other measures assessing common mental health problems was not studied.

### Implications

Given that the present study has developed context specific clinically validated cut-off values, the Sinhala version of the MBI-SS could be used as a screening tool for the assessment of burnout at the school level. However, it is recommended to cross-validate the cut-off values in further studies prior to the widespread utilization of the study instrument. Owing to its brevity, relative ease of administration and sound psychometric properties, this instrument could be effectively used at school settings for screening purposes and to identify students who need the appropriate referral for the management of the problem. Thus, it allows identification of the affected students at early stages, which is important in effective secondary prevention, and identification of vulnerable students, which is imperative for the primary prevention. The present study is one of the few studies conducted with regard to developing clinically validated cut-off values for the MBI-SS, hence, the study findings broaden the global evidence base and signifies the importance of developing context specific cut-off values, rather than using arbitrary cut-off values for assessing burnout as a dichotomous measure in different populations.

## Conclusions

The present study generates the clinically validated cut-off values for the Sinhala version of the 15-item MBI-SS subscale scores as 12.5, 7.5 and 10.5 for the EX, CY and rPE subscales respectively. The diagnostic accuracy of the Sinhala version of the 15-item MBI-SS, using the clinically validated cut-off values and based on the “exhaustion+ 1” criterion, is high.

## Abbreviations

CY: Cynicism
EX: Exhaustion
ICD 10: 10th revision of the International Statistical Classification of Diseases and Related Health Problems
LR-: Negative Likelihood Ratio
LR +: Positive Likelihood Ratio
MBI–SS: Maslach Burnout Inventory-Student Survey
NPV: Negative Predictive Value
PE: Professional Efficacy
PPV: Positive Predictive Value
ROC: Receiver Operating Characteristic
rPE: reversed Professional Efficacy
SD: Standard Deviation
